# Abdominal effusion revealing an exophytic hydatid cyst of the liver has developed under mesocolic

**DOI:** 10.11604/pamj.2019.34.101.20475

**Published:** 2019-10-18

**Authors:** Riyad Abbas, Rabbani Khalid, Louzi Abdelouahed, Finech Bennasser

**Affiliations:** 1 Departement of General Surgery, Arrazi Hospital, Mohammed VI University Medical Center, Marrakech, Morocco

**Keywords:** Hydatic cyst, liver, under mesocolic

## Abstract

The hydatid cyst of the liver is a parasitic disease due to the development of echinococcosis granulosus. It is common in livestock regions in developing countries but is gaining interest in the West due to migratory flows. If it remains a benign and asymptomatic affection for a long time, its natural evolution is often enamelled of complication which can put at the risk of vital prognosis. Diagnosis and staging are based on morphological examinations, including ultrasound and CT scan. The hydatid serology retains a place especially for the detection of recurrence after hydatid cyst of the liver surgery. In addition to surgery considered up to as the radical treatment of choice, other techniques have appeared in the therapeutic arsenal in combination with oral treatment with albendazol for uncomplicated cases thus reducing the morbidity of surgery. We report a case of giant hydatid cyst associated with exophytic liver development under mesocolic associated with a peritoneal hydatidosis.

## Introduction

The hydatic cyst of the liver generates various pathological lesions responsible for various polymorphic clinical pictures; its treatment aims to eliminate the parasite, and to solve the problem of the residual cavity and any associated complications. The therapeutic methods are numerous [1], medical and surgical, by classical or laparoscopic way, but none can be erected in standard gold because of the diversity of the anatomopathological lesions. A hydatid cyst of the liver is a benign parasitic tumor affecting both sexes and all ages, the choice of a method in the therapeutic arsenal available should allow healing with almost no mortality and the lowest possible morbidity, avoiding the risk of recurrence.

## Patient and observation

**Figure 1:**
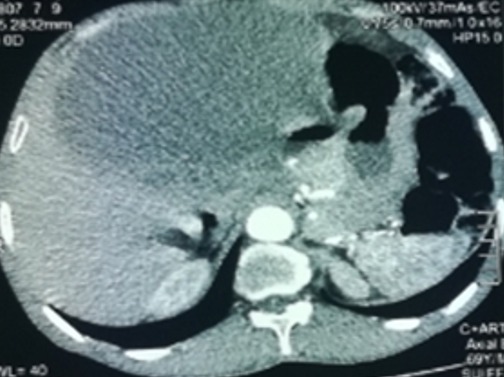
CT image of the hydatid cyst

**Figure 2:**
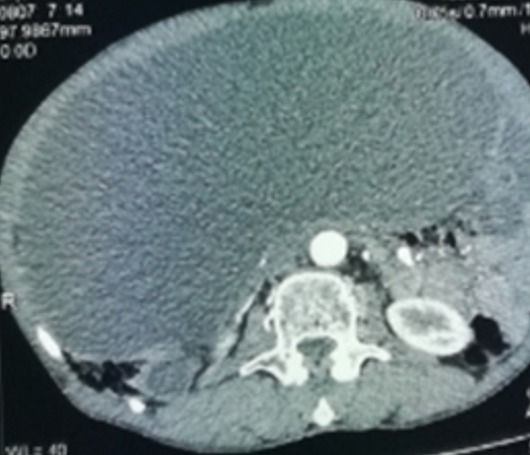
CT image of the hydatid cyst in its under mesocolic portion

**Figure 3:**
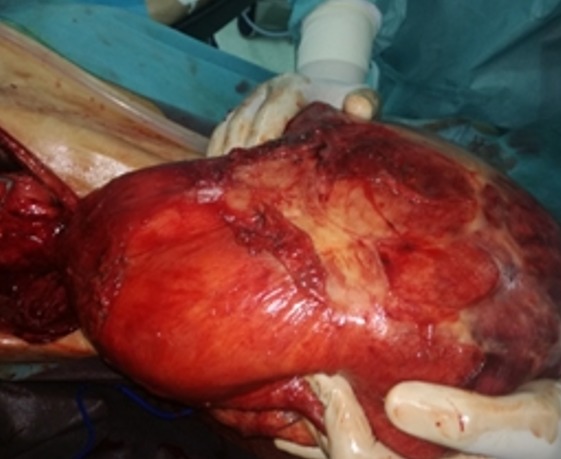
Peroperatory overview

**Figure 4:**
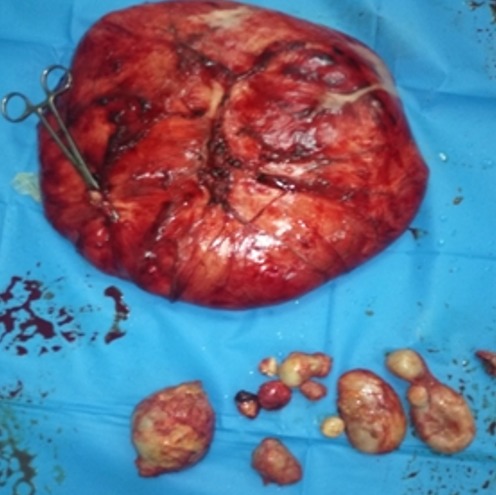
The different resected cysts

This is a 57-year-old patient with no notable pathological history, originally from rural areas. He presented for 12 months of mild abdominal pain and intermittent aggravated by abdominal distention and feeling of heaviness without other associated signs or deterioration of the general condition. Clinically there is abdominal distension with generalized dullness. Apart from a positive serology, the remainder of the biological examination was unremarkable. The ultrasound shows a large heterogeneous abdominal mass whose origin is difficult to determine. A requested abdominal CT scan finds: bulging abdominal fluid formation not elevated measuring 27cm/26cm/16cm seat of parietal calcification and membranes displacing the digestive structures (Figure 1, Figure 2), this formation is associated with multiple hepatic hydatid cysts, in the right hypochondrium and left of different stages. The patient was operated and the exploration found a hydatid cyst of segment III adhered to the gall bladder and extending through a narrow path at the level of the mesocolic stage where it develops to create a huge cystic mass of 30cm long axis (Figure 3), there are also multiple cysts of different size and stage in the liver, small and large epiploon, gastric ligament and right parietocolic gutter (Figure 4). The gesture consisted of a perikystectomy, carrying the cyst in its entirety, for the rest of the hydatid cysts sitting in the liver a resection of the salient dome was made; the other cysts were resected in their entirety.

## Discussion

**Epidemiology:** echinococcosis is an unevenly distributed zoonosis with areas of high endemicity such as the Mediterranean (Maghreb, Greece, Turkey), the Middle East, Latin America (Argentina, Chile) and China [2]. It affects both sexes with a female predominance [3]. Factors favoring Echinococcus granulosus infestation are occupations exposed to contact with livestock and dogs, uncontrolled slaughter and defective hand and food hygiene [3].

### Positive diagnosis

**Clinic:** circumstances of discovery are very diverse, dominated by: pain of the right hypochondrium, right basithoracic, right lumbar or epigastric. Fortuitly during a consultation for other affection, examination of employment, the balance of extension for other localization. Clinically: the abdominal inspection is most often normal. Sometimes there is a curvature of the right hypochondrium. Palpation and percussion seek hepatomegaly: it is inconstant. The hydatid cyst of the liver is sometimes seen as a rounded or oval mass, well limited, firm or renitant, painless, mobile with the liver when breathing.

### Paraclinic

**Abdominal ultrasound:** as a key diagnostic test in view of its availability and reliability [4], it makes it possible to specify the headquarters, the size, the reports and the starification of Gharbi. The classification of Gharbi [5] was the first and most commonly used classification (Table 1). This classification is now increasingly being replaced by that developed by the informal working group on echinococcosis of the World Health Organization. Health (WHO) [2] (Table 2). It is distinguished from the by introducing the notion of "cysticlesion" which is a unilocular lesion, small (often less than 0.5 cm) without its own wall visible and inverting types II and III of both classifications [6] Table 3.

**Table 1:** Gharbi classification

type I	Rounded hypoechoic formation limited by a clean wall and giving a posterior reinforcement. This type corresponds to the uni-vesicular cyst
type II	Hypoechoic rounded formation limited by a clean wall that is peeled off. This type corresponds to the stage of detachment of membranes
type III	Rounded formation hypo-echoic multi-partitioned in "honeycomb". This type corresponds to the multi-vesicular cyst.
type IV	Heterogeneous round formation of pseudo-tumoral appearance. It is very suggestive of hydatid cyst of the liver when it contains serpiginous, "draped" or "bulbous onion" images (corresponding to collapsed vesicles and membranes), or when it also includes small formations hypo-echogenic (corresponding to girls' vesicles). This type corresponds to a cyst with gelatinous content or putty.
type V	Rounded formation with a hyperechoic wall with posterior shadow cone. This type corresponds to a cyst whose percyyst is calcified

**Table 2:** WHO classification of liver hydatid cysts

CL	Univésicular, Cystic lesion (CL) with a Uniform anechoic content, not clearly defined by a hyperechoic margin (= non-visible cyst wall)
CE1	Univésiculaire, simple cyst with uniform anechoic content. Cyst can be à fine echo of the movement of the capsule brood which is often called hydatid sand ('snowflake sign') Wall of the visible cyst
CE2	Multivesicular, multicleaned cysts, septa Ons of the cyst produce a 'wheel + like' structure, and the presence of daughter vesicles is indicated by 'rosette' or 'nidd'abeille' structures. The daughter vesicles may partially or completely occupy the vesicle of the mother cyst. Normally visible cyst wall
CE3	Univésiculaire cyst which can contain girls vesicles. Anechoic content with detachment of a laminated membrane from the visible cyst wall as a floating membrane or as a 'water + lily sign' which is indicative of floating membranes above debris of cystic fluid.
CE4	Degenerative, heterogeneous, hypoechoic or hyperechoic content. No blisters girls. Can show a 'wool ball' sign that indicates degenerative membranes
CE5	Cysts characterized by a calcified thick wall that is arc-shaped, producing a shadow cone. Degree of calcification varies from partial to complete

**CL** = Cystic lesion - **CE** = Cystic Echiniccocus or hydatid cyst

**Table 3:** Correspondence between Gharbi and WHO classification of liver hydatid cysts

Gharbi	WHO
-	CL
Type I	CE1
Type II	CE3
Type III	CE2
Type IV	CE4
Type V	CE5

**Abdominal tomography:** CT is useful when the diagnosis is difficult (Gharbi type I, IV and V) by eliminating differential diagnoses, namely: a biliary cyst, a hepatic angioma, an adenoma, a hepatocarcinoma, a liver abscess or a hepatic metastasis in its cystic form. Computed tomography is also indicated in case of multiple hydatid localization, complicated hydatid cyst of the liver or in case of hydatid recurrence [7].

**Hydatid serology:** it finds its interest in case of doubt diagnosis especially for hydatid cyst of the liver type I and IV classification Gharbi and for detection of recurrence after surgical treatment, it is based on the detection of antiparasitic antibodies present in the blood [2]. Several techniques are used immunoelectrophoresis, ELISA, indirect immunofluorescence, western blot and hemmaglutination. The identification of immunoglobulin subclasses is useful in the early diagnosis of hydatid recurrence and their distinction from primary infections. Thus, IgG4 would be markers of hydatid recurrence whereas IgG2 would be associated with primary infections [8].

### Treatment

**Objectives:** the objectives of the treatment of the hydatid cyst of the liver are the destruction of the parasite, the treatment of the cystic cavity, the treatment of the possible complications and the prevention of the hydatid recurrences.

### Non-operative treatment

**Medical treatment:** oral antihelminthics have a direct effect on scolex and perhaps also on the membrane with reduced permeability [9]. Albendazole (ABZ) is the most commonly used. The combination ABZ-praziquantel would be more effective than the ABZ alone [10]. ABZ is prescribed at a dose of 10 to 15 mg/kg/day in two doses taken orally. Treatment is continuous for 3 to 6 months [11]. Efficacy is monitored on ultrasound, which seeks a decrease in the volume of hydatid cyst of the liver or an increase in the echogenicity of its contents [12]. When ABZ is prescribed in combination with another procedure, it is given 4 days to 1 month before surgery or percutaneous puncture, then 3 months after [9].

**Percutaneous puncture (PAIR):** the percutaneous treatment (PAIR) includes the percutaneous puncture of the cysts by means of an echographic or scanner control, the aspiration of the cystic fluid, the injection of a scolicidal agent for a duration of 10 to 15 min and the re-aspiration of the liquid [13]. The most used scolicidal agents are 25% sodium chloride solutions and 95% alcohol [2]. Standardization of the procedure was made in 2001 by the World Health Organization (WHO) [10]. Percutaneous treatment is contraindicated in the case of cyst type CE2, CE3b, CE4 and CE5. The aspiration of a bilious liquid during the procedure must imperatively indicate the stop of the treatment and the recourse to the surgical treatment [5]. ABZ is prescribed per os before and after the procedure [9]. It is indicated for inoperable patients and those who refuse surgery or in case of hydatid recurrence after surgical treatment [2].

### Surgical treatment

**Which way first?** the classical approach is a right subcostal laparotomy that can be enlarged on the left for cysts of the left liver 2. The laparoscopic approach for the management of hydatid cyst of the liver was first reported in 1992 [14], A retrospective study published in 2014 concluded that the laparoscopic route was the only predictor of hydatid recurrence [15].

**What scholic agent?** the use of formalin and hydrogen peroxide as an agent scolicide was discontinued following the occurrence of accidents and serious complications such as anaphylactic shock, sclerosing cholangitis and air embolism [16]. Currently experts from the World Health Organization (WHO) recommend the protection of the operative field by abdominal compresses soaked with hypertonic saline at 25%, 25% saline intra-cystic injection, before its opening, with a contact time of at least 15 minutes. This procedure should be avoided if there is suspicion of a cyst-biliary fistula [5].

**Radical or conservative surgery?** radical techniques include regulated hepatectomies and total perkystectomy, while conservative surgery can be summarized as partial perkystectomy (resection of the cysts salient dome) [1]. The current data in the literature agree that radical surgery should be preferred whenever possible because it reduces postoperatively the risk of deep abdominal infections, biliary fistulas, global morbidity and recurrence without increasing the postoperative mortality rate [17].

**Prevention:** the fight against hydatidosis cannot be conceived without the implementation of control and prophylaxis measures adapted to the local or regional context concerned [2]: health education programs for exposed populations; rigorous legislation against uncontrolled slaughter ; limitation of stray dog populations; screening and treatment of parasitized dogs; vaccination of livestock remains the domain of research despite concrete progress.

## Conclusion

The hydatid cyst of the liver is a parasitic condition that can remain latent for a long time. The treatment of hydatid cyst of the liver is mainly surgical. However, it remains difficult to codify because of the diversity of pathological forms of the disease. And despite improvements in surgical techniques, high morbidity remains associated with the disease. Efforts in control, health education and prevention need to be stepped up to reduce parasitic transmission.

## Competing interests

The authors declare no competing interests.
